# Two-Year Outcomes of Novel Dual-Mobility Implant in Primary Total Hip Arthroplasty

**DOI:** 10.1155/2024/4125965

**Published:** 2024-01-16

**Authors:** Benjamin C. Schaffler, Hayley E. Raymond, Collin S. Black, Akram A. Habibi, Mallory Ehlers, Stephen T. Duncan, Ran Schwarzkopf

**Affiliations:** ^1^New York University Langone Orthopedic Hospital, New York, NY, USA; ^2^University of Kentucky, Department of Orthopaedic Surgery, Lexington, KY, USA

## Abstract

**Introduction:**

Dual-mobility (DM) implants for total hip arthroplasty (THA) have gained popularity due to their potential to reduce hip instability and dislocation events that may lead to revision surgery. These implants consist of a femoral head articulated within a polyethylene liner, which articulates within an outer acetabular shell, creating a dual-bearing surface. Our study aimed to report our observations on the survivorship of a novel DM implant for primary total hip arthroplasty at two years.

**Methods:**

We conducted a retrospective, multicenter study to assess the clinical outcomes of patients undergoing a THA with a novel DM implant (OR3O acetabular system™, Smith & Nephew, Inc., Memphis, TN) from January 2020 to September 2021. Patient demographics, surgical information, and survivorship data were collected from medical records for patients with a minimum of two years of follow-up. Primary outcomes included overall implant survivorship at two years as well as aseptic survivorship, revision rates of the DM acetabular shell, and average time to revision. Patient-reported outcomes were collected in the form of HOOS JR.

**Results:**

A total of 250 hips in 245 patients had a minimum two-year follow-up. Primary osteoarthritis (80%) was the most common indication for index THA. The average aseptic survivorship of the DM acetabular components at two years for the cohort was 98.4% and survivorship of the acetabular implants overall was 97.6%. There were a total of four (1.6%) aseptic revisions of the DM acetabular component. Reasons for aseptic acetabular revision included one case of instability, one intraprosthetic dislocation, one periprosthetic acetabular fracture, and one malpositioned acetabular cup resulting in impingement. The mean time of follow-up was 893.9 days. Eighty-seven patients had preoperative and two-year HOOS JR available. HOOS JR improved by an average of 38.5 points.

**Conclusion:**

This novel DM acetabular implant demonstrates excellent survivorship at two years follow-up with low rates of instability and intraprosthetic dislocation and no episodes of metal-on-metal corrosion. Use of the DM implant demonstrated clinically relevant improvements in patient-reported outcomes at two years.

## 1. Introduction

Since the first total hip arthroplasty (THA) in 1891, significant changes have been made to the design of the bearing surfaces [1]. Bousquet et al. proposed a concept of dual-mobility (DM) in 1974 to improve hip stability by increasing the femoral head-to-neck ratio [2, 3]. Since it was first proposed, DM liners have gained popularity in the primary and revision hip arthroplasty settings as a potential tool to help prevent instability events [4]. DM articulations consist of a femoral head that is intercalated within a polyethylene liner. This inner liner then articulates with an outer acetabular cup, creating a dual-bearing surface. The majority of motion occurs at the small articulation between the head and the liner with the larger articulation of the liner and shell occurring when the stem's neck contacts the liner [5]. The significantly larger head leads to an increased jump distance, along with an increased impingement-free range of motion of the prosthesis, which contributes to its role in preventing instability and dislocation [6].

Instability has been shown to be the most common reason for revision total hip arthroplasty in the United States, accounting for 23% of all revisions [7]. With the prevalence of total hip arthroplasty continuing to rise and younger patients receiving the procedure, it is increasingly important to address implant stability concerns during the primary procedure [8, 9]. One potential strategy is the expanded use of modular DM implants in patients at elevated risk of instability during primary total hip replacement. While standard ceramic-on-polyethylene or metal-on-polyethylene acetabular liners remain common and the gold standard, the use of DM for primary total hip replacement has grown in popularity. Instability after THA remains a common reason for failure. Certain patient populations, such as those with neurological disease, fused spinal alignment, or significant hip or acetabular deformity, as well as those with certain intraoperative findings, such as abductor deficiency, are at elevated risk of dislocation [5]. Surgeons will often weigh the option of using a DM implant more heavily in these at-risk patients. However, each surgeon's threshold for using a DM implant in patients at high risk for instability varies. In our study, patients included those with an abnormal spinopelvic relationship or neurologic disease or where the surgeon intraoperatively decides for higher stability. DM implants have been reported to have excellent survivorship with low rates of instability and dislocation for primary hip replacement surgery [10]. Although they are not without their own unique concerns, including the risk of intraprosthetic dislocation, metal particle disease, and elevated metal ions secondary to metal-on-metal corrosion, the advent of newer modular DM designs promises that these will remain an excellent option for primary THA in the future [11]. Newer designs have looked to further lower the risk of metal wear in modular DM arthroplasty through the use of novel metal alloys. One such novel design uses an oxidized zirconium niobium alloy in the DM liner design rather than cobalt chrome or titanium alloys, which has been shown to lead to less implant wear elsewhere in arthroplasty [12]. In the present study, we investigated the survivorship at a minimum two-year follow-up of a novel modular DM implant for use in primary THA. Our hypothesis was two-fold. First, we hypothesized that the novel modular DM acetabular implant that uses oxidized zirconium niobium alloy (Oxinium™, Smith & Nephew, Inc., Memphis, TN) in both the inner articulation and the acetabular liner would have good survivorship, demonstrate satisfactory PROMs, and demonstrate a low rate of perioperative complications and revisions comparable to published data regarding other comparative DM designs and fixed-bearing implants that use cobalt chrome or other alloys in their design. Second, we further hypothesized that the use of oxidized zirconium would lead to low rates of metal wear complications.

## 2. Methods

**Figure 1:**
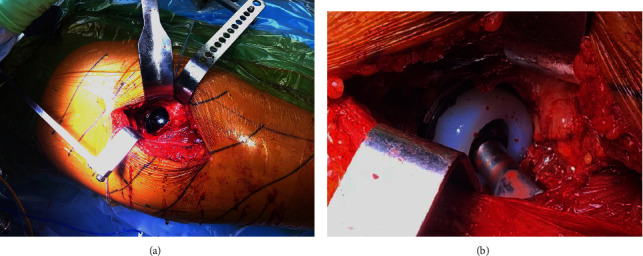
Intraoperative photographs showing the novel DM implant with an Oxinium liner (a) before liner insertion and (b) after final implantation of polyethylene and reduction of the stem.

**Figure 2:**
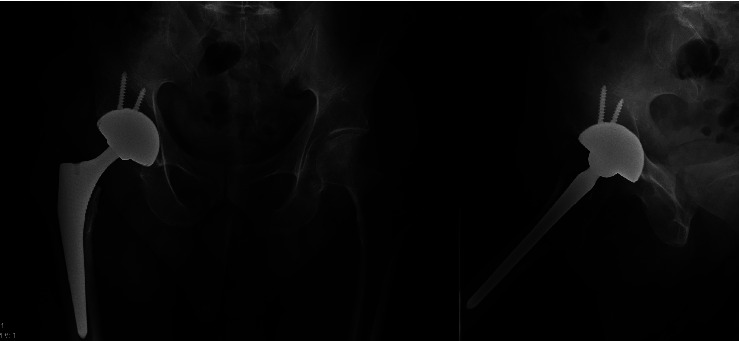
Postoperative AP and lateral radiographs demonstrating implantation and appropriate positioning of the novel DM implant used in primary total hip arthroplasty.

A retrospective, multicenter study was conducted to assess this novel DM system (OR3O acetabular system™, Smith & Nephew, Memphis, TN). Institutional Review Board approval was obtained before the start of the study (IRB #i17-01223). The study included total hip arthroplasties performed by two fellowship-trained surgeons from January 2020 to September 2021. All surgeries were performed through a posterior approach based on surgeon preference and training. All participating surgeons used two acetabular screws routinely as part of acetabular fixation. The joint capsule was routinely repaired if tissue was viable, and the external rotator muscles were routinely repaired to the trochanter via drill tunnels and permanent sutures. All patients included in the study had a minimum of two years of follow-up. All patients in the study received an R3 (Smith & Nephew, Inc., Memphis, TN) press-fit acetabular shell implant and all patients received an Oxinium inner head with the addition of two acetabular screws per institutional practices (Figures 1 and 2). The use of cement or press-fit femoral fixation for the index procedure varied based on patient-specific characteristics and surgeon preference (Table 1). However, all femoral components were from the same manufacturer. Exclusion criteria included patients undergoing revision THA, pregnancy, cases of THA for malignancy, and simultaneous bilateral THA cases.

Patient demographics, initial diagnosis, postoperative complications, and revisions were queried from the institutions' electronic medical records (Epic, Verona, WI). Patient demographics consisted of age, BMI, and sex. Surgical information included index THA approach, indication for index THA, indication for revision THA if applicable, and length of time from index surgery to revision. Primary outcomes were revisions of the DM acetabular shell and liner, indication for revisions, and time from index surgery to revision. Isolated revisions of the femoral stem for femoral periprosthetic fracture were reported, but excluded in the analysis of DM survivorship if revision was limited to the femoral stem without revision of the acetabular shell or conversion to a non-DM articulation. Revisions were classified as all-cause, including all revisions, or aseptic revision, indicating that the revision was performed for a complication other than the periprosthetic joint infection.

Patient-reported outcomes in the form of hip dysfunction and osteoarthritis outcome scores for joint replacement (HOOS JR) were collected as standard-of-care at preoperative and subsequent follow-up visits. These values were reported and analyzed.

Descriptive statistics were performed, including means and standard deviations. Survivorship data analysis, including Kaplan–Meier survival curves, was performed using IBM SPSS (Version 28.0.1.1, Armonk, NY). The sample size justification of 250 hips was based on a post hoc power analysis to contextualize the study's ability to detect a meaningful difference in survivorship rates. With an assumption of an aseptic survivorship of at least 98% as a performance goal and with a 5% noninferiority margin of the lower confidence limit, at least 219 hips would be needed to be evaluated to detect a significant difference at a conservative alpha of 0.025 and  >90% power (Clopper–Pearson exact method), lending confidence to the size of the number of cases reviewed in this study.

## 3. Results

Of the 245 patients who received index THA, 49.2% were male (Table 2). The average age was 61.0 (range: 30 to 85), and the average BMI was 29.51 (range: 16.5 to 52.8) (Table 2). Of the 5 patients who had THA performed on both hips, procedures were staged by a minimum of 3 months. Primary osteoarthritis (80%) was the most common indication for index THA (Table 3). Other indications for index THA included avascular necrosis (9.6%), femoral neck fracture (3.6%), posttraumatic (3.2%), acetabular dysplasia (2.4%), inflammatory arthritis (0.4%), and untreated slipped capital femoral epiphysis (0.4%).

**Figure 3:**
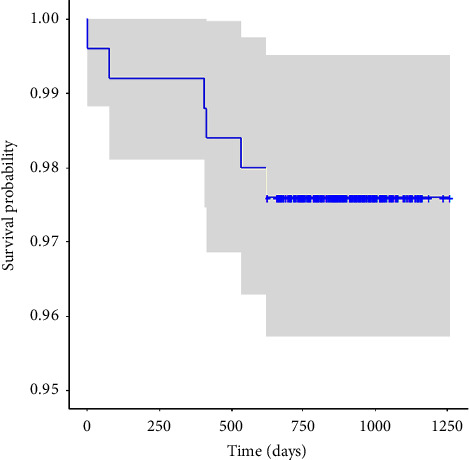
Kaplan–Meier overall acetabular survival rates of a dual-mobility total hip arthroplasty implant.

**Figure 4:**
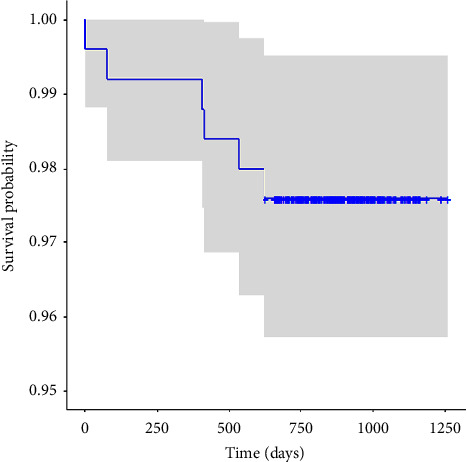
Kaplan–Meier aseptic acetabular survival rates of a novel dual-mobility total hip arthroplasty.

The mean time to follow-up for all patients was 893.9 days. At a minimum of two years follow-up, the aseptic survivorship for the DM acetabular implant was 98.4% (Figure 3). Including infections, the DM acetabular components achieved 97.6% survivorship (Figure 4). There were no cases of metal-on-metal corrosion or metal ion disease reported in the cohort. The all-cause revision rate was 4.4%, which included four isolated femur fractures treated with isolated femoral revision where the DM acetabular cup was retained, which was not included in the DM acetabular implant survivorship. There were a total of four (1.6%) aseptic acetabular revisions. Of these, only one (0.4%) was due to instability (persistent dislocation). One additional patient underwent an acetabular revision for a traumatic intraprosthetic dislocation (0.4%) which resulted in an exchange of the polyethylene liner and femoral head. One patient (0.4%) had their acetabular cup revised for persistent impingement believed to be due to initial cup malpositioning, and one patient (0.4%) experienced a late traumatic periprosthetic acetabular fracture requiring revision of the cup. The septic revision rate was 1.2% (three patients) and included one patient who underwent debridement, antibiotics, and implant retention (DAIR) and two who underwent standard two-stage exchange with initial placement of an antibiotic spacer. The average time to acetabular revision was 342.7 days (Table 4). The average time to aseptic acetabular revision was 338.5 days (Table 4).

A total of 87 patients had reported HOOS JRat both their preoperative and two-year visits. The average HOOS JR at preoperative visits was 48.7 (Table 5). Of patients with reported HOOS JR at two-year follow-up, the average score was 87.2, resulting in an average increase in HOOS JR of 38.5.

## 4. Discussion

In the present study, the use of a novel modular DM implant in the setting of primary THA demonstrated high survival rates at two years, with aseptic revision-free survival of the acetabular components of 98.4%. Additionally, these implants demonstrated low rates of instability and low rates of intraprosthetic dislocation with only one reported case of revision for each. There were no reported cases of metal corrosion resulting in implant failure.

The performance of this modular implant is consistent with the high performance and survivability noted in newer generations of modular DM in the literature. Tarazi et al. noted excellent survivorship and patient-reported outcomes at seven years in patients who received modular DM implants for primary THA [13]. Baker et al. examined five-year outcomes of a modular dual-mobility implant and noted excellent functional outcomes with no modular failure or dislocations with only 3.2 percent of hips needing revision at final follow-up [14]. In a systematic review of all DM hips, Darrith et al. showed a high survival rate of 98% at long-term follow-up (8.5 years) and a low dislocation rate (0.46%), which were both improved compared to cohort-matched patients receiving fixed-bearing implants [15]. Similarly, Jobory et al. compared 4,520 THAs performed with a conventional articulation that were propensity score matched with an equal number of THAs that used modular DM articulations and found that the DM group had a lower risk of revision. They had similar results when looking at revisions only for instability, where the DM group again had lower rates than the conventional implant group [16]. Rowan et al. also performed a matched-cohort analysis of patients under 55 years of age undergoing primary total hip replacement and noted that the DM group had zero dislocations or intraprosthetic dissociations compared to the seven patients in the fixed-bearing group [17]. Our study similarly demonstrated the stability of DM implants with only one case each of instability (0.4%) and intraprosthetic dislocation due to trauma (0.4%). Scott et al. looked specifically at impingement rates and demonstrated that, in a cadaveric retrieval study, DM liners showed less evidence of impingement (21.5%) compared to fixed-bearing liners (77%), although their study did not delineate between whether components were modular or not [6].

DM implants are not without their own complications. The inner liner can dissociate from the femoral head component, otherwise known as an intraprosthetic dislocation. This is unique to DM implants and can happen due to trauma or from closed reduction attempts with excess force on a DM component [18]. Historically, intraprosthetic dislocation would occur following polyethylene wear, but the advent of newer-generation polyethylene liners and DM components has made this exceedingly rare, and most intraprosthetic dislocations with new implants occur as a result of attempted closed reduction or trauma [19]. Rates of intraprosthetic dislocation in the literature are reported at only 2-3% at 15-year follow-up. However, they are serious in that they require open reduction and revision of polyethylene liner or full component revision to correct [20]. The novel implant studied in this paper had a low rate of intraprosthetic dislocations, with one patient experiencing a traumatic intraprosthetic event, for a rate of 0.4% in the cohort. This is consistent with other DM implants commonly in use today and had comparable/superior outcomes in two-year survivorship, all-cause revisions, and instability events compared to other DM components [21].

The articulation between two dissimilar metals in arthroplasty can result in mechanical corrosion, generation of metal ions leading to foreign body reaction, and ultimately implant failure. As modular DM implants have risen in popularity, there have been reports of metal-on-metal failure between the inner and outer acetabular shells [11]. Kolz et al. performed a small retrieval study in which they noted evidence of corrosion in 12 cobalt-chromium liners [22]. Hemmerling et al. followed up with a similar retrieval study of 60 DM components and noted microscopic evidence of fretting in 88% and corrosion in 97% [23]. Similar findings have been reported in titanium modular components as well [24]. This has led to a focus on novel modular DM designs using harder metals such as zirconium, or ceramicization of the articulation between components, to lessen the potential for metal-on-metal wear. The novel DM implant in the present study, which is designed with oxidized zirconium, did not experience any issues of mechanical-assisted corrosion failure in the present study. Additionally, the implant in this study is built with an inner liner taper angle of 18 degrees, which has been shown to lead to improved rates of malseating [25].

HOOS JR collected in one-third of patients improved by an average of 38.5 points at two years with patients on average reporting a score of 87.2 at two-year follow-up. HOOS JR are a quick and easy patient-reported outcome measure that show high consistency and validity than other hip scoring tools [26]. The patient's acceptable symptom state, a measure of acceptable outcome after surgery, for HOOS JR has been calculated as 76.7, meaning that on average the patients receiving primary THA with the novel DM implant surpassed that threshold, signifying good PROM outcomes [27]. These findings are corroborated by those of other studies, where DM implants in the setting of primary THA yield high PROMS. Singh et al. compared primary THA patients who received a fixed-bearing, monoblock or modular DM implant and found no difference in PROMs at two years in the DM groups [28]. Tarazi et al. also noted excellent Harris hip scores (HHS) at long-term follow-up (seven years) in patients who received a primary THA with DM liner [13].

This study is not without its limitations. Due to reporting and patient participation, we only have patient-reported outcome measures for one-third of our patient population and the study's conclusions would benefit from having outcomes of a larger cohort. Additionally, our follow-up only goes out to two years. Given the average lifespan of a total hip replacement and the aging population, the study would benefit from longer-term follow-up. It is our intention to continue following this cohort for five and potentially ten years to better report on longer-term outcomes.

## 5. Conclusion

In this study, we report on two-year outcomes of a novel modular DM implant for primary total hip arthroplasty. In 250 patients undergoing primary THA, the novel DM implant achieved high aseptic survivorship at two years (98.4%) and the rate of aseptic revision of the DM acetabular component was 1.6%. The DM implant performed well from an instability standpoint and had low rates of DM-specific complications including intraprosthetic dislocation and modular metal corrosion and led to clinically relevant improvements in patient-reported outcomes. These results show that this implant is an effective and safe choice for use in the primary total hip setting and demonstrates good survivorship with a low complication rate.

## Figures and Tables

**Table 1:** Femoral stem fixation type for study patients, stratified by age and gender.

	Male	Female	Average age (years)
Cementless	83	88	59
Cemented	13	13	67

**Table 2:** Baseline patient demographics (*n* = 250 hips in 245 patients).

Demographic	No. (%)
Mean age, yrs (SD)	61.0 (10.8)
*Sex, no.* (%)	
Male	122 (49.8%)
Female	123 (50.2%)
Mean body mass index, kg/m^2^ (SD)	29.5 (6.5)

**Table 3:** Indications for index total hip arthroplasty with a dual-mobility implant.

Indication for index THA	No. (%)
Osteoarthritis	201 (80.4%)
Avascular necrosis	24 (9.6%)
Femoral neck fracture	9 (3.6%)
Posttraumatic	8 (3.2%)
Dysplasia	6 (2.4%)
Inflammatory	1 (0.4%)
Slipped capital femoral epiphysis	1 (0.4%)

THA, total hip arthroplasty.

**Table 4:** Time to acetabular revision surgery after total hip arthroplasty with a dual-mobility implant.

	Acetabular-specific revision	Aseptic acetabular-specific revision
Days to revision surgery (mean (SD))	342.7 (227.0)	338.5 (200.6)

**Table 5:** Patient-reported outcome measures after total hip arthroplasty with a dual-mobility implant.

Time after surgery	Preoperative (*n* = 95)	Two weeks (*n* = 43)	Six weeks (*n* = 68)	One year (*n* = 56)	Two years (*n* = 87)
HOOS JR (mean (SD))	48.7 (19.4)	66.5 (16.0)	80.2 (17.1)	78.8 (14.5)	87.2 (16.6)

HOOS JR, hip dysfunction and osteoarthritis outcome scores for joint replacement.

## Data Availability

The survivorship and patient-reported outcomes data used to support the findings of this study are available from the corresponding author upon request.
